# PTMint database of experimentally verified PTM regulation on protein–protein interaction

**DOI:** 10.1093/bioinformatics/btac823

**Published:** 2022-12-22

**Authors:** Xiaokun Hong, Ningshan Li, Jiyang Lv, Yan Zhang, Jing Li, Jian Zhang, Hai-Feng Chen

**Affiliations:** State Key Laboratory of Microbial Metabolism, Joint International Research Laboratory of Metabolic & Developmental Sciences, Department of Bioinformatics and Biostatistics, National Experimental Teaching Center for Life Sciences and Biotechnology, School of Life Sciences and Biotechnology, Shanghai Center for Systems Biomedicine, Shanghai Jiao Tong University, Shanghai 200240, China; Department of Bioinformatics and Biostatistics, SJTU-Yale Joint Center for Biostatistics and Data Science, School of Life Sciences and Biotechnology, Shanghai Jiao Tong University, Shanghai, 200240, China; State Key Laboratory of Microbial Metabolism, Joint International Research Laboratory of Metabolic & Developmental Sciences, Department of Bioinformatics and Biostatistics, National Experimental Teaching Center for Life Sciences and Biotechnology, School of Life Sciences and Biotechnology, Shanghai Center for Systems Biomedicine, Shanghai Jiao Tong University, Shanghai 200240, China; State Key Laboratory of Microbial Metabolism, Joint International Research Laboratory of Metabolic & Developmental Sciences, Department of Bioinformatics and Biostatistics, National Experimental Teaching Center for Life Sciences and Biotechnology, School of Life Sciences and Biotechnology, Shanghai Center for Systems Biomedicine, Shanghai Jiao Tong University, Shanghai 200240, China; State Key Laboratory of Microbial Metabolism, Joint International Research Laboratory of Metabolic & Developmental Sciences, Department of Bioinformatics and Biostatistics, National Experimental Teaching Center for Life Sciences and Biotechnology, School of Life Sciences and Biotechnology, Shanghai Center for Systems Biomedicine, Shanghai Jiao Tong University, Shanghai 200240, China; Key Laboratory of Cell Differentiation and Apoptosis of Chinese Ministry of Education, Department of Pathophysiology, Shanghai Jiao-Tong University School of Medicine (SJTU-SM), Shanghai 200025, China; State Key Laboratory of Microbial Metabolism, Joint International Research Laboratory of Metabolic & Developmental Sciences, Department of Bioinformatics and Biostatistics, National Experimental Teaching Center for Life Sciences and Biotechnology, School of Life Sciences and Biotechnology, Shanghai Center for Systems Biomedicine, Shanghai Jiao Tong University, Shanghai 200240, China

## Abstract

**Motivation:**

Post-translational modification (PTM) is an important biochemical process. which includes six most well-studied types: phosphorylation, acetylation, methylation, sumoylation, ubiquitylation and glycosylation. PTM is involved in various cell signaling pathways and biological processes. Abnormal PTM status is closely associated with severe diseases (such as cancer and neurologic diseases) by regulating protein functions, such as protein–protein interactions (PPIs). A set of databases was constructed separately for PTM sites and PPI; however, the resource of regulation for PTM on PPI is still unsolved.

**Results:**

Here, we firstly constructed a public accessible database of PTMint (PTMs that are associated with PPIs) (https://ptmint.sjtu.edu.cn/) that contains manually curated complete experimental evidence of the PTM regulation on PPIs in multiple organisms, including *Homo sapiens*, *Arabidopsis thaliana*, *Caenorhabditis elegans*, *Drosophila melanogaster*, *Saccharomyces cerevisiae* and *Schizosaccharomyces pombe*. Currently, the first version of PTMint encompassed 2477 non-redundant PTM sites in 1169 proteins affecting 2371 protein–protein pairs involving 357 diseases. Various annotations were systematically integrated, such as protein sequence, structure properties and protein complex analysis. PTMint database can help to insight into disease mechanism, disease diagnosis and drug discovery associated with PTM and PPI.

**Availability and implementation:**

PTMint is freely available at: https://ptmint.sjtu.edu.cn/.

**Supplementary information:**

Supplementary data are available at *Bioinformatics* online.

## 1 Introduction

Post-translational modification (PTM) is an important biochemical process among several organisms. There are over 400 known PTM types, of which six types are well studied, including phosphorylation (Phos), acetylation (Ac), methylation (Me), sumoylation (Sumo), ubiquitylation (Ub) and glycosylation (Glyco). Most biological process and signaling pathway occur by interaction of two or more proteins (De Las Rivas and Fontanillo, 2010), which are regulated by PTM (Seet *et al.*, 2006). Abnormal PTM status on proteins could lead to severe diseases [such as Alzheimer's disease (Lau *et al.*, 2008), cancer (Gu *et al.*, 2013) and cardiovascular disease (Coxon *et al.*, 2012)] by regulating protein functions, such as protein–protein interactions (PPIs).

Thousands of PTM sites of several organisms have been identified owing to the advancements in mass spectrometry (MS) (Choudhary and Mann, 2010; Gao and Chen, 2021; Swietlik *et al.*, 2020). The abundant PTM data are stored in publicly available resources, such as PhosphoSitePlus (Hornbeck *et al.*, 2015), qPhos (Yu *et al.*, 2019), Phospho.ELM (Dinkel *et al.*, 2011), dbPTM (Li *et al.*, 2022), PLMD (Xu *et al.*, 2017) and the universal database, Uniprot (UniProt, 2012). However, the role of massive PTM sites in protein, especially on the PPIs is still remained to clarify.

Uniprot database offers the ‘PTM/Processing’ section to record the PTM sites and/or processing events. However, its relatively hard for inexperienced users to search and browse, and it also lacks the information of PTM onto the 3D structures. Another database, PhosphoSitePlus (Hornbeck *et al.*, 2015) provides PTM effects on PPIs based on literature mining with Linguamatics software (Bandy *et al.*, 2009), which inevitably includes much false positive results. To date, several tools and resources are emerged to predict the PTM functions. PTMfunc predicts the PTM effect based on the conservation in the domain (Beltrao *et al.*, 2012). PTMcode provided known and predicted functional associations between PTMs based on co-evolution theory (Minguez *et al.*, 2015). Another two different methods, one is Mechismo web server based on interface pair potentials (Betts *et al.*, 2015) and the other is FoldX software based on empirical forcefield (Schymkowitz *et al.*, 2005), which rely on interfacial PTM sites in practice, solely.

With all the above in consideration, we presented the PTMint database, a comprehensive experimentally verified PTM effects on PPIs, such as PTM types and sites, interaction proteins, detection methods, associated diseases and co-localization. Moreover, in order to facilitate the investigation of PTM roles, we combined the experimental evidence with sequence and structure annotation. This database will be helpful for researchers to explore the relationship among PTM, PPIs and diseases in sequence and structure aspects.

## 2 Materials and methods

### 2.1 Data sources

**Fig. 1.**
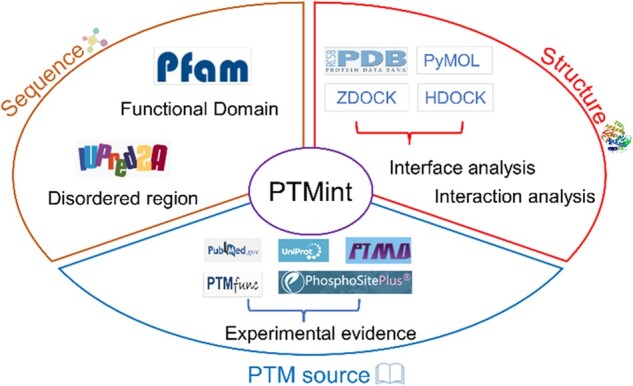
The overall design and construction of PTMint database

The workflow of the PTMint database construction was shown in Figure 1, including data collection and annotation. We defined the regulatory roles of PTM on PPIs (Betts *et al.*, 2015, 2017; Li *et al.*, 2012; Lin *et al.*, 2021; Seet *et al.*, 2006; Spektor and Rice 2009; Wang *et al.*, 2022): (i) Enhance: Increase affinity and (ii) Inhibit: Decrease affinity. We extracted the functional PTM sites and associated literature from Uniprot (UniProt, 2012), PTMD (Xu *et al.*, 2018), PTMfunc (Beltrao *et al.*, 2012) and PhosphoSitePlus (Hornbeck *et al.*, 2015) databases. We also downloaded the relevant literature using PubMed database by searching the following keywords and the combinations: *Homo sapiens*, *Arabidopsis thaliana*, *Caenorhabditis elegans*, *Drosophila melanogaster*, *Saccharomyces cerevisiae*, *Schizosaccharomyces pombe*, protein, bind, associate, enable, interact, interaction, inhibit, disable, prevent, dissociate, site, PTM, Phos, Ac, Me, Sumo, Ub and Glyco. Then, we checked the full text of the above nearly 3600 papers carefully to obtain complete experimental evidence, which included regulatory PTM sites and types, interacting proteins, detection methods, associated diseases and co-localization. Briefly, we examined the relationship between PTM and disease based on cellular or animal disease models in each literature. Then, based on the detection method of PPIs, we examined the protein interactions affected by PTM in the full text. By the way, we established the relationship among PTM, protein interactions and diseases.

### 2.2 Protein sequence analysis

We searched the Uniport database (UniProt, 2012) to obtain all canonical protein sequences. And the sequence window (upstream and downstream five residues around the PTM sites) was also extracted. The disorder propensity scores were calculated by IUPred2A (Meszaros *et al.*, 2018). Protein sequences were annotated using Pfam database to obtain functional domain information (Mistry *et al.*, 2021).

### 2.3 Protein structure

For individual proteins, we downloaded the full-length protein structures in bulk from AlphaFold Protein Structure Database (AlphaFold DB) (Varadi *et al.*, 2022). If the protein length was longer than 2700 amino acids, we used AlphaFold (version 2.1.2) (Jumper *et al.*, 2021) to predict the domain structures of long-length proteins, respectively. For protein complexes, all the paired protein sequences were mapped to the PDB database (Berman *et al.*, 2000) using blastp against pdbaa with e-value of 10^−4^. The PDB entries were selected according to the following criteria: (i) The PTM sites existed on the structures. (ii) The protein name of matched sequence was same as query protein. (iii) The matched complexes with two chains were preferred. Due to the limited crystal structures, a large scale of protein–protein dockings was performed by molecular docking softwares. ZDOCK is a fast Fourier transform-based docking procedure for rigid proteins that searches for all possible binding modes in the translational and rotational space between two proteins and evaluates each pose using an energy-based scoring function (Pierce *et al.*, 2014). HDOCK is the hybrid docking algorithm of template modeling and free docking based on the docking program and allows the users to provide possible protein–protein binding sites to perform rapid protein–protein docking (Yan *et al.*, 2020). PyMOL performs molecular docking based on template alignment, which maximizes the retention of the docking mode of the original template structure (DeLano, 2010). For homology modeling, we used PyMOL (version 2.4.1) to obtain complex structure based on PDB template structure. For molecular docking, we used ZDOCK (version 3.0.2) and HDOCK (version 1.1) by using XL-MS data (cross-linking) through exhaustive curation of published literatures and predicted domain–domain interactions provided by INstruct database (Meyer *et al.*, 2013) as the docking constraints. For the protein–protein docking results provided by all softwares, we uniformly used the structure with the highest score in the software as the final docking complex structure.

### 2.4 Interaction analysis

Interaction assignment was handled with in-house software (Chen and Luo, 2007; Wang *et al.*, 2014). The hydrophobic interaction (HP) is defined when the mass centers of side chain for hydrophobic residues are closed within 6.5 Å. The charge–charge interaction within 11 Å plays a key role in protein/ligand-binding free energy (Qin *et al.*, 2010). Thus, the distance between the mass centers of charge residues is less than 11 Å, which was considered as electrostatic interaction (ELE). A hydrogen bond (HB) within the complex is defined when the distance of two polar heavy atoms is less than 3.5 Å and the bond angle is larger than 120°. We utilized the InterfaceResidues.py Python script created by Vertrees J (https://pymolwiki.org/) for complex interfaceResidues analysis. Briefly, this Python script splits the complex into two pieces for two interacting chain and then calculates the difference between the complex-based accessible surface areas and the chain-only-based accessible surface areas. If the value is greater than cutoff (the default is 1.0 Å2), the residues is marked as interfacial residue. The same process was handled for PTM sites to label interfacial PTM sites.

### 2.5 Secondary structure analysis

To obtain the property of the secondary structure of complex structures and PTM sites, the secondary structure content was calculated by Dictionary of Protein Secondary Structure algorithm (Kabsch and Sander, 1983) according to the residue-specific HBs in eight secondary structures (π-helix, 3, (10)-helix, α-helix, β-bridge, β-sheet, turn, bend and coil). For simplification, we have classified into four types: (i) Helix: π-helix, 3, (10)-helix and α-helix; (ii) Sheet: β-bridge and β-sheet; (iii) Turn: turn; (iv) Loop: bend and coil.

### 2.6 Score calculation

Among the 20 basic amino acids in proteins, some amino acids are frequently PTM-modified, such as Serine (S), Threonine (T), Tyrosine (Y), Lysine (K) and Arginine (R). Serine and Threonine can be modified by Phos and Glyco. And Lysine can be modified by multiple modifications, such as Ac, Me, Sumo and Ub. To assess the important regulatory role of PTM in PPI networks *in vivo*, we introduced the importance score of PTM sites.

This importance score takes into account of the number of PTM types and interacting proteins and calculated with Equation (1).

(1) Score=2 • ∑(PTM • N).

In which N is the number of protein which specific PTM site regulates.

And the Score can be normalized with Equation (2).

(2) $$\begin{array}{c} \text{Normalized}\_\text{score}=1-1/\text{Score}\\ \text{Normalized}\_\text{score}\in \left[0.5,1\right) \end{array}.$$

The normalized_score reflects the relative importance of specific regulatory PTM sites, which will increase as the number of PTM types or interaction proteins increase.

### 2.7 Database and web interface implementation

The web interfaces were implemented in Hyper Text Markup Language (HTML), JavaScript (JS) and Cascading Style Sheets. And the web frame was supported by Bootstrap v4 framework. Furthermore, 3Dmol.js plugin was employed to visualize protein 3D structures (Rego and Koes, 2015). And the PPI network was analyzed and visualized by ECharts plugin (Li *et al.*, 2018). Besides, all figures and tables in the website were performed in Python.

## 3 Results

### 3.1 Database and content

**Fig. 2.**
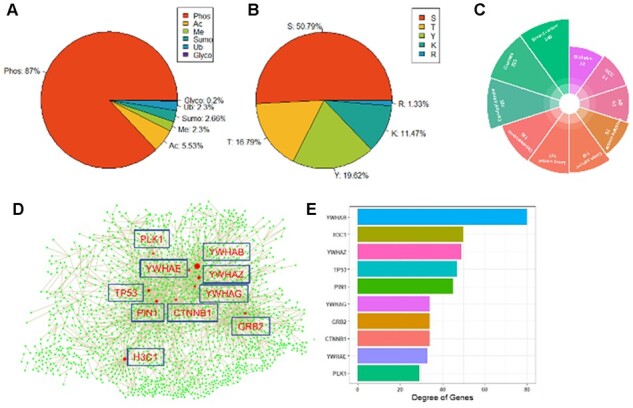
The data in the PTMint database. Statistical analysis results in terms of PTM types (**A**) and PTM sites (**B**). (**C**) Top 10 diseases affected by PTM. (**D**) PTM-associated PPI network. Top 10 genes with highest degrees were highlighted in PPI (D) and visualized in bar plot (**E**)

The current version of PTMint contains 2477 non-redundant PTM sites in 1169 proteins affecting 2371 protein–protein pairs involving 357 diseases. Uniport database provides the 425 records of PTM effect on PPI and 322 functional PTM sites. PTMD database provides the 45 records of PTM effect on PPI and 34 functional PTM sites. And PhosphositePlus guides us to search some reference literature based on text mining. In the two regulatory roles (Enhance and Inhibit), ‘Enhance’ has a bigger proportion, suggesting that PTM might tend to increase PPIs (Table 1). In our results, the top 1 of six main PTM types with the largest number is Phos (87%) (Fig. 2A). The main regulatory PTM sites are Serine (S), Threonine (T) and Tyrosine (Y), which have a cumulative proportion of 87.20% (Fig. 2B).

**Table 1.** Summary of PTM effects on PPIs

PTM types	PTM sites	Enhance	Inhibit
Phosphorylation	S, T, Y	2157	968
Acetylation	K	170	86
Methylation	K, R	81	19
Sumoylation	K	53	26
Ubiquitylation	K	60	11
Glycosylation	S, T	5	0

As shown in Supplementary Table S1, of all the experimentally verified regulatory PTM sites, 15.82% localizes in the protein–protein interfaces, suggesting a large proportion of non-interfacial sites can also regulate molecular interactions; 36.61% can be found in the functional domains, implicating the important biological role of PTM in modulating protein function. In the view of secondary structure, PTM sites tend to localize in the loop region rather than structured regions (Helix, Sheet and Turn) (Table S2). Based on results of score calculation, the score of most PTM sites (fraction: 74.54%) is 0.5, which indicates most site modified by one type of PTM, can modulate one PPI in the collected data. And the K10 site of H3C1 possesses the highest score of 0.97. Ac of K10 can regulate five different PPI, including BAZ1B, BRD7, CHD4, CRH and TRIM33. In addition, the Me of K10 can regulate 14 different PPIs, including AGO3, CBX1, CBX3, CDYL, CDYL2, CHAMP1, CHD4, DCAF8, HSFY1, KAT5, MAD2L2, POGZ, UHRF1 and ZNF470.

To further understand the intrinsic characteristics of PTM-modified proteins and interactor proteins, these all proteins were grouped into multiple classifications according to the biological function (Supplementary Table S3), mainly enzymes and transporters, suggesting these proteins with PTM participate in extensive biological processes and signaling pathways. In the database, there are total 2960 complex structures which 360 structures (fraction: 12.16%) come from PDB experimental structures, 203 structures (fraction: 6.86%) from homology modeling (PyMOL) and 2397 (fraction: 80.98%) structures from molecular docking (ZDOCK and HDOCK). According to the prior docking knowledge (XL-MS and domain–domain interaction), each complex was assigned a confidence value (High, Medium or Low), 32.26% of which were ‘High’ or ‘Medium’.

The top 10 diseases affected by PTM were counted and shown in Figure 2C, which mainly included following three types: (i) cancers: breast cancer (number: 340), cancers (number: 323), cervical cancer (number: 302), osteosarcoma (number: 136), lung cancer (number: 127), colon cancer (number: 119), prostate cancer (number: 76) and hepatocellular carcinoma (HCC) (number: 61); (ii) Alzheimer's disease (AD) (number: 63); and (iii) diabetes (number: 52). We also constructed a PTM-associated PPI network (Fig. 2D). Nodes with high degree in PPI network represent the core nodes. The results showed that the top 10 genes with the highest degrees were: YWHAB (degree: 80), H3C1 (degree: 50), YWHAZ(degree: 49), TP53 (degree: 47), PIN1 (degree: 45), GRB2 (degree: 34), YWHAG (degree: 34), CTNNB1 (degree: 34), YWHAE (degree: 33) and PLK1 (degree: 29) (Fig. 2D and E), suggesting that these genes were disease-susceptible and potential drug targets.

### 3.2 Web search function

**Fig. 3.**
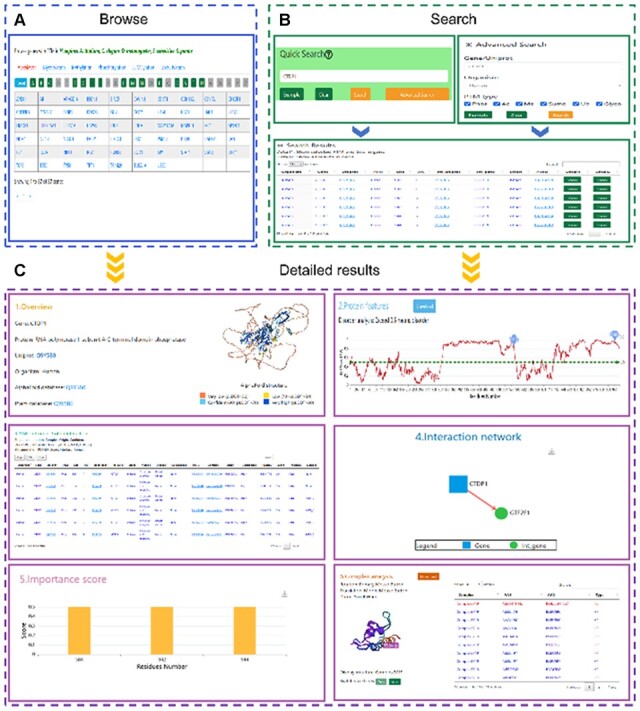
The detailed information in PTMint database. (**A**) Browse function. (**B**) Search_function. (**C**) The returned search results using the CTDP1 as an example. The entry page consists of six major sections: (1) Protein overview. (2) Protein features. (3) PTM on PPIs. (4) Interaction network. Blue square: gene, Green circle: Int_gene, Red arrow: from gene to int_gene interaction. (5) Importance score of PTM sites and (6) complex analysis (A color version of this figure appears in the online version of this article)

Quick search and advanced search were implemented on the homepage and ‘Search’ page, respectively. On the homepage, the user can directly search the database by inputting keyword (such as Gene, Uniprot, PTM, Effect and Organism) (Fig. 3B). Single or multiple filter conditions, such as Gene/Uniprot, Organism and PTM types can be specified on the ‘Search’ page (Fig. 3B). Taking ‘CTDP1’ gene as an example, the searched results will be shown in a tabular format, including Organism, Gene, Uniprot, PTM, Site, AA, Int_uniprot, Int_gene, Effect, PMID and Detail (Fig. 3B). Hyperlinks for Uniprot and PubMed are provided. And the two types of detailed results can be shown by clicking the ‘Show’ buttons, respectively (Fig. 3B).

The typical result page consists of six main sections (Fig. 3C), including protein overview (such as organism, protein name, protein structure and protein domain information), protein features (disorder analysis), PTM on PPIs, interaction network, importance score of PTM sites and complex analysis. In ‘PTM on PPIs’ section, complete experimental evidence and structure, site annotation were integrated (such as whether PTM site localized on the interface or protein domain, complex origin), which can be saved or searched, easily. In ‘Complex analysis’ section, complex structures came from PDB database (Berman *et al.*, 2000) and local molecular dock. And PTM site and type were mapped onto the structure. And users can easily manipulate and switch structures. Furthermore, protein interactions (HB, HP and ELE) calculated by in-house software (Chen and Luo, 2007; Wang *et al.*, 2014) are shown in a tabular format. InterfaceResidues was also calculated to annotate the spatial location of PTM sites. ‘Download’ function was provided for users to download protein features and all complex information composed of complex structures, interfaceResidues and interaction. We also provided several external links, such as Uniport database (UniProt, 2012), AlphaFold database (Varadi *et al.*, 2022), Pfam database (Mistry *et al.*, 2021) and PubMed database by clicking underlined links.

### 3.3 Web browse function

The PTM types and genes were sorted and organized in alphabetical order, which allow the user to quickly obtain interested results (Fig. 3A).

### 3.4 Web download and help function

All data in the PTMint database can be downloaded in the ‘Download’ page, including PTM experimental evidence and protein structure information. And detailed instructions were available in the ‘Help’ page.

## 4 Discussion

To our knowledge, PTMint database is the first comprehensive database of experimental evidence of the PTM effects on PPIs, which not only includes complete experimental records, such as PTM types and sites, interacting proteins, detection methods, associated diseases and co-localization, but also integrates the according sequence and structure annotation (such as molecular dock and interaction analysis), systematically.

PTM level in proteins is controlled precisely based on a temporal and spatial context (Cohen, 2002; Hunter, 1995). And the same site might have different PTM types in various physiological states, such as cancer and hypoxia (Xu *et al.*, 2022). For example, Lysine (K) can selectively be acetylated, methylated or ubiquitylated. Serine (S) can be phosphorylated or glycosylated. In addition, we found the specific PTM type in a site of the protein, can regulate several proteins in our collected data. For example, phos-S289 in MDM4 can simultaneously induce MDM4-MDM2 and MDM4-p53 interactions (Wu *et al.*, 2012). Beta2 integrin Phos on Thr758 acts as a molecular switch to inhibit filamin binding and enhance the 14-3-3 protein binding to the integrin cytoplasmic domain (Takala *et al.*, 2008). Furthermore, 14-3-3 proteins, which contain a phosphoprotein-binding domains (PPBDs), can bind phos-T32 FOXO3 (Singh *et al.*, 2010), phos-S253 FOXO3 (Singh *et al.*, 2010), phos-T642 TBC1D4 (Ramm *et al.*, 2006), phos-S939 TSC2 (Cai *et al.*, 2006), phos-S981 TSC2 (Cai *et al.*, 2006) and phos-S99 BAD (Polzien *et al.*, 2009) in the PI3K-Akt signaling pathway. In order to assess the roles of regulatory sites, all regulatory sites of the protein are ranked according to the importance score, which calculated by PTM types and protein counts. Higher score means higher important role in disease process and potential drug targets. For example, the Y654 of CTNNB1 has a high score of 0.93, inhibiting its Phos by Imatinib offered a therapeutic value in patients with chronic myeloid leukemia (CML) (Coluccia *et al.*, 2007), which was in accord with PTM-associated PPI network results (Fig. 2D and E).

According to previous reports (Betts *et al.*, 2017; Shi *et al.*, 2001; Song *et al.*, 2008), PTM sites which located in the interface between two proteins, can regulate protein interactions. We supposed whether PTM sites which not located in the interface, can also enhance or inhibit interactions. Therefore, we analyzed all protein complex and interface amino acids. To our surprise, both interfacial and non-interfacial sites possess the regulatory roles (Supplementary Table S1). For example, phos-Y47 in Fe65 which not localizes in the interface, decreased Fe65 and RASD1 affinity by distal regulation (Lau *et al.*, 2008). Two PTM sites might be associated with cross-talk pattern based on spatial proximity (Brooks and Gu, 2003; Christensen *et al.*, 2005; Minguez *et al.*, 2015), so cooperation and antagonism among several PTM sites in two interacting proteins could be investigated by above labeled spatial location. Moreover, owing to above collected experimental evidence and structural annotation, a high-fidelity machine learning prediction method considering interface information and local microenvironment (Lu *et al.*, 2022) [such as partial charges, spatial location of carbon (C atom), nitrogen (N atom), oxygen (O atom), hydrogen (H atom) and sulfur (S atom), and solvent accessibility], which assessing PTM (such as Phos, Ac, Me, Sumo, Ub and Glyco) effects (‘Enhance or ‘Inhibit’) on PPI (Betts *et al.*, 2015; Schymkowitz *et al.*, 2005) can be developed in the future. Although several algorithms has been developed to predict kinase-specific Phos sites (Wang *et al.*, 2020; Xue *et al.*, 2005), to predict Phos sites that specifically interact with phosphoprotein-binding domains (Guo *et al.*, 2020), a machine learning method to predict the PTM sites that govern PPIs in the view of PTM position, motif length and residues weights based on the sequence window we provided (upstream and downstream five residues around the PTM site) could be a promising and challenge work.

Due to the limited crystal structures, several software and webserver offer the solution by molecular dock, such as ZDOCK (Pierce *et al.*, 2014), HDOCK (Yan *et al.*, 2020), ClusPro (Kozakov *et al.*, 2017) and HADDOCK (van Zundert *et al.*, 2016). In order to ensure the accuracy of docking results, a large scale of molecular docking was performed combined with experimental XL-MS data (cross-linking) and predicted domain–domain interactions. Furthermore, we also analyzed the interaction (HB, HP and ELE) to help researchers to better understand the PTM roles in disease. Changes of interactions (such as HB, HP and ELE) and structure (such as allostery, disorder-to-order, electrostatic potential and dynamic correlation network) induced by PTM can explain why PTM could regulate PPIs and disease progression (Devanand *et al.*, 2018; Lingyun and Ji-Bin, 2017). Therefore, our database will provide a valuable structural basis for further investigations, such as molecular dynamics simulation (MD) and Markov state models (MSMs). Furthermore, development of a specific forcefield for the simulation of protein–protein complex (Piana *et al.*, 2020) modified by multiple PTM types could be a valuable research filed. Although the statistical results were obtained based on our collected data (Table 1, Supplementary Tables S1–S3), but it might be potentially biased due to the limited PTM types and well-studied proteins.

PTMint database can be further improved in the following aspects. First, the current version of the database contains six main model organisms and PTM types. More organisms and PTM types will be added. Second, additional curations, such as PTM-targeted drugs, PTM expression analysis, PTM-associated survival analysis and association between PTM and mutation will be integrated. Third, we will replace the original predicted complex structures when newly high-resolution complex structures are released in the PDB database. In the future, we will continually maintain and update the PTMint database, when newly regulatory PTM sites are reported in the literature.

In conclusion, we developed PTMint, a comprehensive database of experimentally verified PTM effects on PPIs. We believed that this database should be a useful platform for biologists and bioinformaticians to explore PTM roles on disease development, diagnosis and drug discovery.

## Supplementary Material

btac823_Supplementary_Data

## Data Availability

All data in PTMint are freely accessible at https://ptmint.sjtu.edu.cn/.
