# Supplementary material for: PTMint database of experimentally verified PTM regulation on protein–protein interaction
Source: Bioinformatics. 2022 Dec 22;39(1):btac823. doi: 10.1093/bioinformatics/btac823 (PMC9848059; doi:10.1093/bioinformatics/btac823)
Supplement: btac823_Supplementary_Data [file btac823_supplementary_data.doc]

Supplementary Information

**PTMint Database of Experimentally Verified PTM Regulation on Protein-Protein Interaction**

Xiaokun Hong^1^, Ningshan Li^2^, Jiyang Lv^1^, Yan Zhang^1^, Jing Li^1,*^, Jian Zhang^3,*^, and Hai-Feng Chen^1^,*

^1^State Key Laboratory of Microbial metabolism, Joint International Research Laboratory of Metabolic & Developmental Sciences, Department of Bioinformatics and Biostatistics, National Experimental Teaching Center for Life Sciences and Biotechnology, School of Life Sciences and Biotechnology, Shanghai Center for Systems Biomedicine, Shanghai Jiao Tong University, Shanghai, 200240, China.

^2^SJTU-Yale Joint Center for Biostatistics and Data Science, Department of Bioinformatics and Biostatistics, School of Life Sciences and Biotechnology, Shanghai Jiao Tong University, Shanghai 200240, China.

^3^Department of Pathophysiology, Key Laboratory of Cell Differentiation and Apoptosis of Chinese Ministry of Education, Shanghai Jiao-Tong University School of Medicine (SJTU-SM), Shanghai 200025, China.

* To whom correspondence should be addressed. Tel: 86-21-34204073 Fax: 86-21-34204073; Email: haifengchen@sjtu.edu.cn; jian.zhang@sjtu.edu.cn; jing.li@sjtu.edu.cn

Table S1. Number of PTM sites located in protein-protein interfaces or functional domain

| PTM Types | Protein-Protein interface | Functional domain |
| --- | --- | --- |
| Phosphorylation | 595 | 756 |
| Acetylation | 45 | 75 |
| Methylation | 26 | 20 |
| SUMOylation | 9 | 18 |
| Ubiquitylation | 20 | 38 |
| Glycosylation | 0 | 0 |

Table S2. Number of PTM sites located in secondary structure regions

| PTM Types | Helix | Sheet | Turn | Loop |
| --- | --- | --- | --- | --- |
| Phosphorylation | 605 | 228 | 230 | 2707 |
| Acetylation | 87 | 26 | 25 | 169 |
| Methylation | 8 | 41 | 4 | 98 |
| SUMOylation | 23 | 5 | 5 | 49 |
| Ubiquitylation | 31 | 7 | 7 | 32 |
| Glycosylation | 0 | 0 | 0 | 5 |

Table S3. Data statistics for PTM-modified proteins and Interactor proteins

| Classification | PTM-modified proteins | Interactor proteins |
| --- | --- | --- |
| Oxidoreductases | 27 | 28 |
| Transferases | 89 | 100 |
| Kinases | 121 | 111 |
| Hydrolases | 111 | 107 |
| Lyases | 4 | 1 |
| Isomerases | 8 | 11 |
| Ligases | 7 | 3 |
| Transporters | 110 | 133 |
| Transcription factors | 28 | 19 |
| Proteases | 7 | 4 |
| Other proteins | 657 | 602 |
